# Supplementary material for: Comparison of dosimetric effects of MLC positional errors on VMAT and IMRT plans for SBRT radiotherapy in non-small cell lung cancer
Source: PLoS One. 2022 Dec 1;17(12):e0278422. doi: 10.1371/journal.pone.0278422 (PMC9714892; doi:10.1371/journal.pone.0278422)
Supplement: Attachment — Submitted filename: 1-Response to Reviewers.docx [file pone.0278422.s001.docx]

Dear Editors:

Many thanks for your letter and the reviewers’ comments concerning our manuscript entitled “Comparison of Dosimetric Effects of MLC Positional Errors on VMAT and IMRT plans for SBRT Radiotherapy in Non-small Cell Lung Cancer”. These comments are all valuable and very helpful for revising and improving the manuscript. We have revised the manuscript according to your kind advices and the reviewer’s suggestions. The main revisions and responses to the reviewer’s comments are as follows:

**Editor:**

**Journal Requirements:**

When submitting your revision, we need you to address these additional requirements.

1. Please ensure that your manuscript meets PLOS ONE's style requirements, including those for file naming. The PLOS ONE style templates can be found at

<https://journals.plos.org/plosone/s/file?id=wjVg/PLOSOne_formatting_sample_main_body.pdf> and <https://journals.plos.org/plosone/s/file?id=ba62/PLOSOne_formatting_sample_title_authors_affiliations.pdf>

**Reply:** Thank you for your suggestion. We have modified it according to the requirements.

2. Please provide additional details regarding participant consent. In the ethics statement in the Methods and online submission information, please ensure that you have specified what type you obtained (for instance, written or verbal, and if verbal, how it was documented and witnessed). If your study included minors, state whether you obtained consent from parents or guardians. If the need for consent was waived by the ethics committee, please include this information.

**Reply:** Thank you for your suggestion. We have included an ethics statement in the methods section, as detailed in lines 53-55 of the manuscript. Oral informed consent was obtained by telephone and the transcript has been uploaded in the ethics statement.

3. Please note that PLOS ONE has specific guidelines on code sharing for submissions in which author-generated code underpins the findings in the manuscript. In these cases, all author-generated code must be made available without restrictions upon publication of the work. Please review our guidelines at https://journals.plos.org/plosone/s/materials-and-software-sharing#loc-sharing-code and ensure that your code is shared in a way that follows best practice and facilitates reproducibility and reuse. New software must comply with the Open Source Definition

**Reply:** Thank you for your suggestion. It was requested that the code be shared. And it can be accessed through the following DOI path: https://doi.org/10.6084/m9.figshare.21342072

4. Thank you for stating in your Funding Statement:

"JD was supported by grants from the Health research Program of Shaanxi Provincial Health Commission (No. 2022D037)."

Please provide an amended statement that declares *all* the funding or sources of support (whether external or internal to your organization) received during this study, as detailed online in our guide for authors at http://journals.plos.org/plosone/s/submit-now. Please also include the statement “There was no additional external funding received for this study.” in your updated Funding Statement.

Please include your amended Funding Statement within your cover letter. We will change the online submission form on your behalf.

**Reply:** Thank you for your suggestion. The requested changes were made, and the revised fund statement was incorporated into the cover letter. The content is as follows: “This work was supported by grants from the Health Research Program of Shaanxi Provincial Health Commission (No. 2022D037). There was no additional external funding received for this study.”

5. In your Data Availability statement, you have not specified where the minimal data set underlying the results described in your manuscript can be found. PLOS defines a study's minimal data set as the underlying data used to reach the conclusions drawn in the manuscript and any additional data required to replicate the reported study findings in their entirety. All PLOS journals require that the minimal data set be made fully available. For more information about our data policy, please see http://journals.plos.org/plosone/s/data-availability.

Upon re-submitting your revised manuscript, please upload your study’s minimal underlying data set as either Supporting Information files or to a stable, public repository and include the relevant URLs, DOIs, or accession numbers within your revised cover letter. For a list of acceptable repositories, please see http://journals.plos.org/plosone/s/data-availability#loc-recommended-repositories. Any potentially identifying patient information must be fully anonymized.

Important: If there are ethical or legal restrictions to sharing your data publicly, please explain these restrictions in detail. Please see our guidelines for more information on what we consider unacceptable restrictions to publicly sharing data: http://journals.plos.org/plosone/s/data-availability#loc-unacceptable-data-access-restrictions. Note that it is not acceptable for the authors to be the sole named individuals responsible for ensuring data access.

We will update your Data Availability statement to reflect the information you provide in your cover letter.

**Reply:** Thank you for your suggestion.

We have shared our raw data and the minimal dataset at：

<https://doi.org/10.6084/m9.figshare.21366744.v1>

Our institute's ethics committee can be reached at the following address:

Shaanxi Provincial Cancer Hospital, Xi’an, Shaanxi, People’s Republic of China

Tel：+86-029-85276017

6. Please include your full ethics statement in the ‘Methods’ section of your manuscript file. In your statement, please include the full name of the IRB or ethics committee who approved or waived your study, as well as whether or not you obtained informed written or verbal consent. If consent was waived for your study, please include this information in your statement as well.

**Reply:** Thank you for your suggestion. The full ethical statement is included in the methods section, on lines 53-55 of the article.

**Reviewer # 1**

1. General recommendations:

To bring simulated results of this work more into context, I suggest that the authors examine the frequency and magnitude of their own truebeam MLC errors. This would show if experimental results here would actually be of use clinically or for QA purposes. If authors are unable to do so, please consider the citations that are currently in the work about frequency and magnitude of MLC error (Chow et al). When authors recommend that errors must be less than a certain amount in lines 173, this assumes that a systematic shift to all MLCs must exist. Please indicate based on citations or your own clinical values if this situation could occur and/or how it would be controlled or monitored.

**Reply:** Thanks for your comments. The purpose of this article is to discuss the sensitivity of MLC errors to PTV and OAR doses in clinical SBRT plans. Our research can help doctors and physicians recognize the relationship between MLC errors and clinical doses. Furthermore, we can provide MLC tolerance specific to SBRT as a treatment reference. According to Oliver et al., there was a linear relationship between various types of MLC errors and PTV gEUD. Table5 shows how the slope parameter from the linear fitting was used to determine the dose (gEUD) sensitivity (%/mm) to MLC position errors. The findings enable us to propose tolerances on MLC leaf positions that are dosimetrically equivalent to those considered acceptable for dose deviations in conventional therapy [2% from TG40 / TG142], at least for the target.

2. Tables in this work do not contain consistent significant digits or units of dose. Please address.

**Reply:** Thank you for the reminder. We have already corrected.

In Fig 3, the unit of variation for CI, GI, and D2cm is %, which has been modified in Fig 3.

In Table 1, 2, 3, and 4, the unit of the gEUD variation is %, which has been modified in the Table 1, 2, 3, and 4.

In Table 5, the dose sensitivity is in units of %/mm.

3. Do clinicians or physicists at your clinic use gEUD to make clinical decisions? How is gEUD a useful metric to monitor for QA? Please address.

**Reply:** Physicists at our clinic use gEUD cost functions for plan optimization, it can be an effective tool for dose sculpting in IMRT/VMAT planning. The generalized EUD can be used to evaluate dose distributions in the tumor and surrounding critical structures. It reports the generalized mean value of the non-uniform dose distribution, which represents the homogenous dose distribution that produces the same local control as that obtained with an inhomogeneous dose distribution for the case of tumors. Moreover, gEUD is closely related to normal tissue complication probability (NTCP). The gEUD is based on the radiobiologic response of the tumor rather than physical dose information, allowing it would be a better predictor of clinical outcome.

The following references provide a more detailed explanation of *gEUD*. (Niemierko A. Reporting and analyzing dose distributions: a concept of equivalent uniform dose. Med Phys 1997;24(1):103-10. doi:10.1118/1.598063. PMID:9029544). This literature has been cited in the article on lines 96 and 97.

4. Please indicate how random errors were simulated? No information is provided. What distribution was used to create random errors? Please cite the following paper in your discussion, as it also simulates random and systematic errors and investigates dosimetric impact: <https://aapm.onlinelibrary.wiley.com/doi/full/10.1002/acm2.12677>

**Reply**: Thanks for your comments.

In this work, each open leaf position in each control point was modified by a number generated from a Gaussian distribution centered on zero, with a standard deviation equal to the error magnitude. Six magnitudes of random error were simulated. To avoid potential errors and unintentional duplication, we generate a new Gaussian random distribution for each error plan, so that the same errors are not added to different exposure fields and plans.

In line 74 and 75, we have provided additional explanations.

5. Specific comments:
1) Abstract:

“The aim of this study was to investigate the difference between MLC positional error and dosimetry of volume intensity modulated (VMAT) and general intensity modulated (IMRT) plans for non-small cell lung cancer (NSCLC).”

The usage of the word “difference” is incorrect here and meaning is not correct.

Instead: The aim of this study was to investigate the impact of MLC positional error on dosimetry of volume intensity modulated (VMAT) and general intensity modulated (IMRT) plans for non-small cell lung cancer (NSCLC)

**Reply：** Thank you for the reminder. We have already corrected it.

2) Lines 225

That all leaves would be systematically shifted by these amounts is very unlikely. The Varian Truebeam monitors leaf positions many times over per second and prevents a beam on if leaves are out of tolerance.

**Reply：** Thanks for your comments. In this work, regarding the dosimetric sensitivity of the PTV and OARs, we implicitly assumed that treatment plans were affected by MLC position alone(line 223). The results were based on this assumption.

3) Line 227-228

“In particular, VMAT plans have a greater impact on dose distribution than IMRT plans and should be carefully monitored”.

The authors should be more specific. The MLC positions are already closely monitored and recorded in the dynalog files. Please indicate what is meant. How specifically do reviewers plan to use these results to guide clinical QA measures? Do the authors mean IMRTQA? Is this work part of a commissioning process, determination of baseline, or educational in nature?

I am of the opinion that this study is very useful for teaching of medical physicists and demonstrating how different MLC errors can impact a patients treatment (if these errors were to not be caught by MLC control system).

**Reply：**Thanks for your comments. The tolerance of the MLC controller is a user-defined parameter. The optimal tolerance value is tight enough to prevent severe dosimetric changes but clinically practical enough to avoid unnecessary treatment session lengthening due to beam delivery interruptions. The purpose of this work was to evaluate the dosimetric impact of both random and systematic errors in the leaf positions of MLC, rather than to perform a retrospective study of the actual leaf position. TG-142 and ESTRO 2008 Reports recommend defining MLC positioning accuracy with qualitative weekly and quantitative monthly QA. For MLC QA, such as EPID(2D) or ArcCheck(3D) can be used for analysis to quantitatively assess MLC positioning errors, according to the results in this work, a rationalization recommendation can be made for MLC accuracy requirements to provide the accurate delivery of IMRT and VMAT.

4) Line 230

“In this study, we simulated that the MLC errors may be within a certain range.”

This sentence is not correct. Instead it should read that “in this study, we simulated MLC errors within a certain range and their dosimetric impact on SBRT plans.”

**Reply:** Thank you for the reminder. We have already corrected.

5) Line 231

“In future research, it would be useful to conduct experimental studies to determine the typical long term accuracy and precision of the relevant parameters for SBRT delivery”

What relevant parameters do the authors mean? This sentence is not specific. Authors need to please recognize that relevant measures that quantify dosimetric impact of MLC errors is a well-studied sub field of medical physics, so what is the authors motivation for continued monitoring?

**Reply:** According to the results of this paper, the dose deviations caused by errors in VMAT plans are greater than those in IMRT plans, so quality control of VMAT has received more attention and monitoring than IMRT. I agree with your assessment and recommendation. The main purpose of this research is to conduct a study of the relationship between MLC errors and clinical geud dosimetry in order to establish clinical criteria for each MLC error tolerance. The previous expression was not entirely accurate. To avoid misunderstanding, this sentence has been removed from the manuscript.

We tried our best to improve the manuscript according to the advices. And we hope that the revision will meet with your approval. Once again, thanks very much for your comments and suggestions. If you have any questions, please contact us at the following address.

Sincerely yours,

Jia Deng

Address:

Department of Radiation Oncology, Shaanxi Provincial Cancer Hospital, Xi’an, Shaanxi, People’s Republic of China

Tel: +8618192331016

E-mail: dengjia92@yeah.net
